# Prediction of repurposed drugs for treating lung injury in COVID-19

**DOI:** 10.12688/f1000research.23996.2

**Published:** 2020-08-26

**Authors:** Bing He, Lana Garmire

**Affiliations:** 1 Department of Computational Medicine and Bioinformatics, Medical School, University of Michigan, Ann Arbor, 48105, USA

**Keywords:** COVID-19, SARS-CoV-2, lung injury, ACE2, COL-3, CGP-60474

## Abstract

**Background:** Coronavirus disease (COVID-19) is an infectious disease discovered in 2019 and currently in outbreak across the world. Lung injury with severe respiratory failure is the leading cause of death in COVID-19, caused by severe acute respiratory syndrome coronavirus 2 (SARS-CoV-2). However, there still lacks efficient treatment for COVID-19 induced lung injury and acute respiratory failure.

**Methods:** Inhibition of angiotensin-converting enzyme 2 (ACE2) caused by the spike protein of SARS-CoV-2 is the most plausible mechanism of lung injury in COVID-19. We performed drug repositioning analysis to identify drug candidates that reverse gene expression pattern in L1000 lung cell line HCC515 treated with ACE2 inhibitor. We confirmed these drug candidates by similar bioinformatics analysis using lung tissues from patients deceased from COVID-19. We further investigated deregulated genes and pathways related to lung injury, as well as the gene-pathway-drug candidate relationships.

**Results:** We propose two candidate drugs, COL-3 (a chemically modified tetracycline) and CGP-60474 (a cyclin-dependent kinase inhibitor), for treating lung injuries in COVID-19. Further bioinformatics analysis shows that 12 significantly enriched pathways (P-value <0.05) overlap between HCC515 cells treated with ACE2 inhibitor and human COVID-19 patient lung tissues. These include signaling pathways known to be associated with lung injury such as TNF signaling, MAPK signaling and chemokine signaling pathways. All 12 pathways are targeted in COL-3 treated HCC515 cells, in which genes such as RHOA, RAC2, FAS, CDC42 have reduced expression. CGP-60474 shares 11 of 12 pathways with COL-3 and common target genes such as RHOA. It also uniquely targets other genes related to lung injury, such as CALR and MMP14.

**Conclusions:** This study shows that ACE2 inhibition is likely part of the mechanisms leading to lung injury in COVID-19, and that compounds such as COL-3 and CGP-60474 have potential as repurposed drugs for its treatment.

## Abbreviations

COVID-19: coronavirus disease 2019, SARS-CoV-2: severe acute respiratory syndrome coronavirus 2, ACE2: angiotensin-converting enzyme 2, AGER: advanced glycosylation end-product specific receptor, LBP: lipopolysaccharide binding protein, SCGB1A1: secretoglobin family 1A member, SFTPD: surfactant protein D, RAS: renin–angiotensin system, Ang II: angiotensin II, Ang-(1-7): angiotensin (1-7), ARDS: acute respiratory distress syndrome, ACE2i: inhibition of ACE2, NS: not significant, NA: not available.

## Introduction

Coronavirus disease 2019 (COVID-19) is an infectious disease discovered in 2019 and currently in outbreak across the world, resulting in more than 4.3 million infections and over 291,354 deaths as of 12 ^th^ May, 2020. It is causing tens of thousands of new infections and thousands of mortalities every day. Patients with COVID-19 present with respiratory symptoms. Severe viral pneumonia related lung injury with acute respiratory failure is the main reason for COVID-19 related death ^1^. However, there still lacks efficient treatment for COVID-19 induced lung injury and acute respiratory failure.

Coronaviruses (CoVs), are a large family of enveloped, positive-sense, single-stranded RNA viruses, which can be found in many vertebrates, such as birds, pigs and humans, and cause various diseases. A novel CoV, termed severe acute respiratory syndrome (SARS)-CoV-2, is the cause of COVID-19. Lung injury with acute respiratory failure was also the main reason for death in patients with SARS ^2^. The spike protein of SARS-CoV-2 shares 79.5% sequence identity with the SARS-CoV virus ^3– 5^, which caused the SARS pandemic in 2002, resulting in 774 deaths in 8096 confirmed patients in 29 countries ^6^. SARS-CoV-2 uses angiotensin-converting enzyme 2 (ACE2) as the entry receptor and cellular serine protease TMPRSS2 for S protein priming to allow fusion of viral and cellular membranes ^7^, similar to SARS-CoV ^8, 9^. Since in SARS-CoV infection, the spike protein of SARS-CoV inhibits ACE2 to cause severe lung injury and acute respiratory failure ^10, 11^, it is highly likely that SARS-CoV-2 uses the same mechanism. Inhibition of ACE2 may be part of the pathogenic mechanism in SARS-CoV-2 induced lung injury and acute respiratory failure. Therefore, a drug repurposing pipeline aiming to reverse the gene expression pattern due to ACE2 inhibition may be a candidate for treating lung injury in COVID-19.

Towards this goal, we performed drug repositioning analysis to identify drugs and compounds for treating SARS-CoV-2 induced lung injury. To explore the mechanisms of proposed drug treatment, we further investigated deregulated genes and pathways in both human lung cells treated with ACE2 inhibitor and human lung tissues from patients deceased from COVID-19. Our results revealed that lung injury related molecular mechanisms are shared between ACE2 inhibition and SARS-CoV-2 infection. Moreover, our proposed drugs can target key genes in these mechanisms, and therefore may prevent lung injury in COVID-19.

## Methods

### Data preparation

RNA-Seq data from human lung tissues from two COVID-19 deceased patients and age-matched healthy lung tissues, as well as human lung A549 cells with or without H1N1 infection, were downloaded from Gene Expression Omnibus (GEO) database (accession number: GSE147507), as reported by Melo *et al.* ^12^. Level 5 LINCS L1000 data, a collection of gene expression profiles for thousands of perturbagens at a variety of time points, doses, and cell lines, were downloaded from the GEO database (accession numbers: GSE70138 and GSE92742). Gene expression profiles in lung cells were extracted from the downloaded L1000 dataset using R scripts (code is available on GitHub) ^13^. The extracted data include 37,366 treatments of 12,706 drugs in 13 lung cell lines at different time points and doses. Two lung cell lines, A549 and HCC515, were treated with 10 µM moexipril, a homologue of ACE2 that inhibits ACE2 and ACE. Gene expression profiles were collected from A549 and HCC515 cells at six and 24 hours after treatment. Upon moexipril treatment, ACE2 level decreased with time in HCC515 as expected; however, levels increased in A549. This prompted us to focus the analysis on the HCC515 line, which showed the inhibition effect of moexipril. Differential expression of genes was measured by z-score ^14^.

### Gene and pathway analysis

The RNA-Seq data were analyzed using DESeq2 ^15^ (version: 1.26.0). Differential gene expressions were identified by comparing cases and controls (e.g. COVID-19 lung tissue vs. the healthy lung tissue, or cells with H1N1 infection vs. those without H1N1 infection). The top 1000 differential expressed genes were selected by the absolute z-score value. These genes were then used for pathway enrichment analysis using Database for Annotation, Visualization and Integrated Discovery (DAVID) v6.8 ^16^. Significant pathways (P-value <0.05) were compared between HCC515 cells with ACE2 inhibitor inhibition and lung tissues from COVID-19 deceased patients. A gene is called “consistent”, if it shows changes in the same direction (increase or decrease) with ACE2 inhibitor treatment and SARS-CoV-2 infection. There are 5390 genes deregulated in the same direction in both ACE2 inhibition-treated HCC515 cell line and COVID-19 patient lung tissue. Among them, 797 genes are in top 1000 differentially expressed genes in either ACE2 inhibition-treated HCC515 cell line or COVID-19 patient lung tissue, and 119 genes are in significantly enriched pathways. The importance of pathways was ranked using the following score:

$$Score_{Pathway}=\sqrt{-log\frac{Pvalue_{ACE2i}+Pvalue_{COVID19}}{2}}n_{consistent}$$

*Pvalue _ACE2i_* is the P-value from pathway enrichment analysis for the top 1000 differentially expressed genes in HCC515 cells treated with ACE2 inhibitor. *Pvalue _COVID19_* is the P-value from pathway enrichment analysis for the top 1000 differentially expressed genes in human lung tissue infected by SARS-CoV-2. *n _consistent_* is the number of consistent genes in that pathway among top 1000 differential expressed genes for both HCC515 cells with ACE2 inhibitor treatment and lung tissues of deceased COVID-19 patients.

The importance of genes was ranked by the following score:

$$Score_{Gene}=\sqrt{\frac{{{\mathrm{Zscore}}_{\mathrm{ACE}2i}}^{2}+{{\mathrm{Zscore}}_{\mathrm{COVID}19}}^{2}}{2}}$$

*Zscore _ACE2i_* is the z-score of the gene in HCC515 cells treated with ACE2 inhibitor. *Zscore _COVID19_* is the z-score of the gene in human lung tissue infected by SARS-CoV-2.

### Drug repositioning analysis

The differential gene expression list was transformed into a gene rank list. An effective drug treatment is one that reverts the aberrant gene expression in disease back to the normal level in health. DrInsight Package ^17^ (version: 0.1.1) was used for this purpose, and the outlier-sum (OS) statistic was retrieved, which models the overall disease-drug connectivity by aggregating disease transcriptome changes with drug perturbation. The Kolmogorov–Smirnov (K-S) test was then applied to the OS statistic, to show the significance level of one drug treatment relative to the background of all other drugs and compounds in the reference drug dataset. The reference drug dataset contains gene rank lists from 12,706 drug treatments in the LINCS L1000 data, as mentioned above. The Benjamini-Hochberg (BH) false discovery rate (FDR) was used to adjust P-values from the K-S test to avoid false significance due to multiple comparisons. FDR<0.05 was used as the threshold to select significant drug candidates for the disease.

### Figure preparation

**Figure 1.**
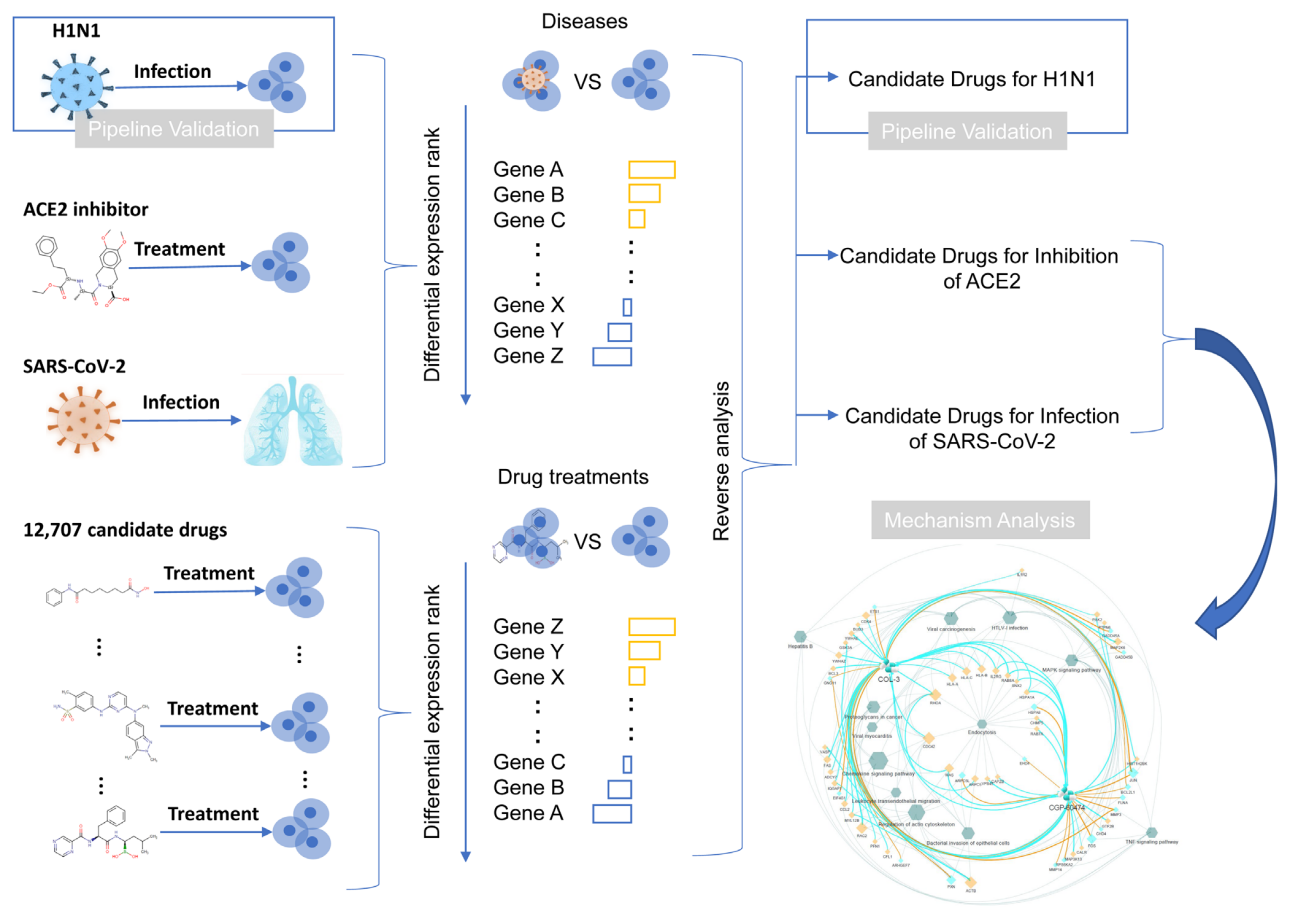
Workflow of repurposing drugs for treating lung injury in COVID-19. Input data include gene expression in A549 cells with H1N1 infection, HCC515 cells with ACE2 inhibitor (ACE2i), human lung tissues of deceased COVID-19 patients and cells with drug treatment. Reversing analysis is conducted to search for drugs that can reverse the gene expression changes upon treatment. The candidate drug to is compared to all other drugs and compounds, in order to estimate its significance level at treating the disease. Candidate drugs for H1N1 are used for validation of the computational pipeline. Candidate drugs identified in both HCC515 cells treated with ACE2 inhibitor and in human lung tissues of deceased COVID-19 patients are used for downstream mechanism analysis.

**Figure 2.**
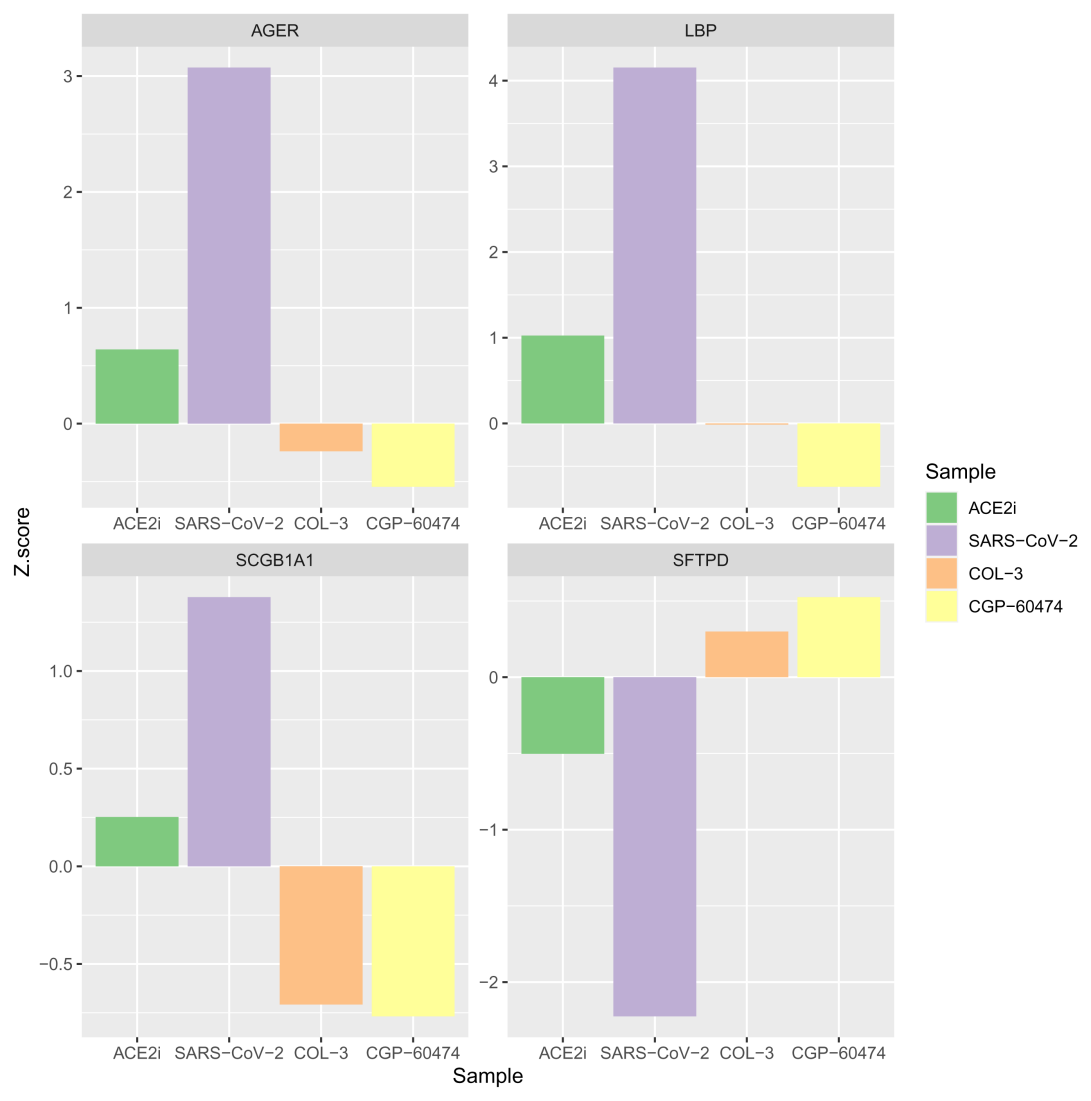
COL-3 and CGP-60474 can reverse the expression of marker genes of lung injury. Z-score: z score of differential expression of genes in the sample; ACE2i: HCC515 cells with ACE2 inhibitor inhibition; SARS-CoV-2: human lung tissues from COVID-19 patients deceased from SARS-CoV-2 induced lung complications; COL-3: HCC515 cells treated with COL-3; CGP-60474: HCC515 cells treated with CGP-60474.

**Figure 3.**
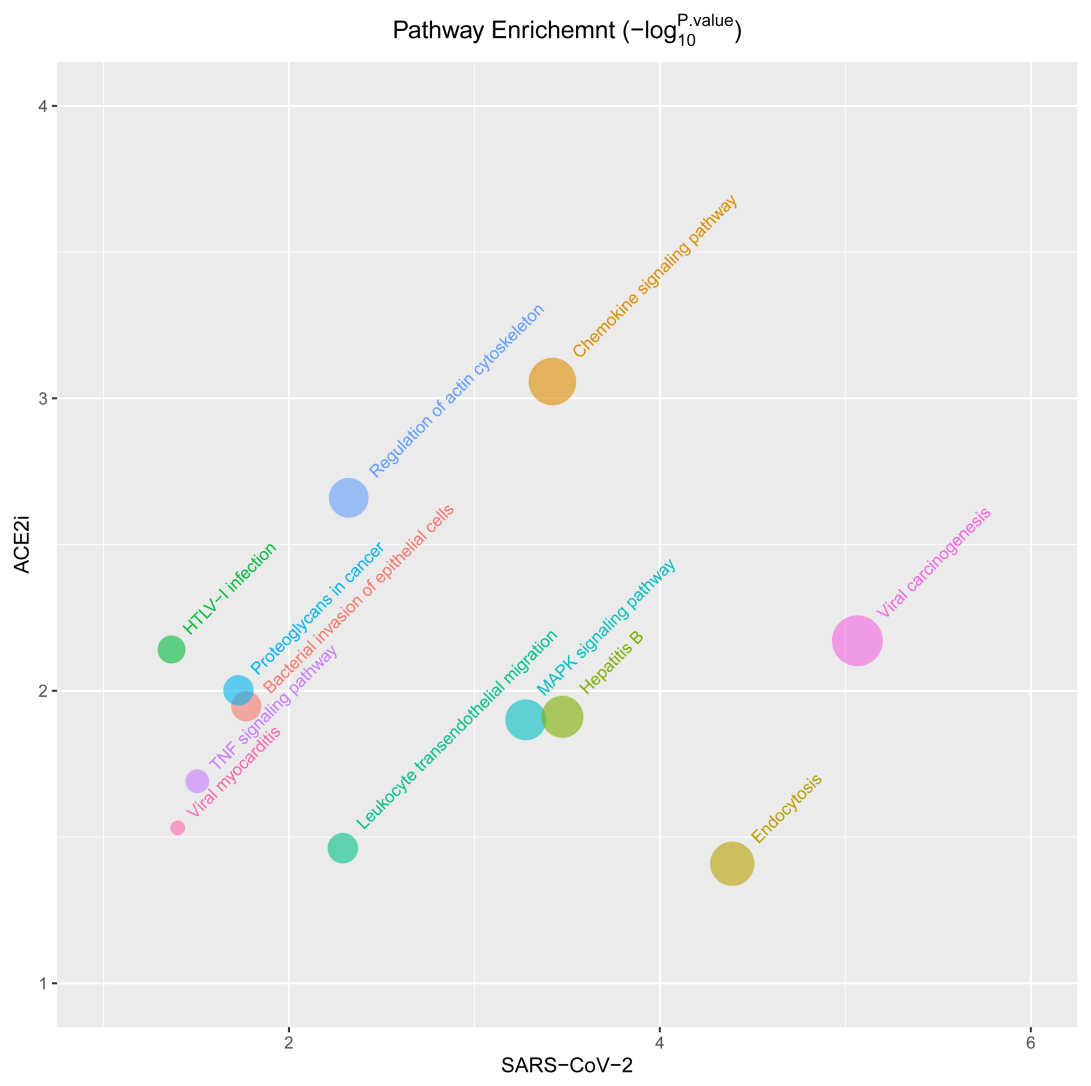
The bubble plot of significantly enriched pathways in HCC515 cells with ACE2 inhibitor inhibition and human COVID-19 patient lung tissues. X-axis and Y-axis show -log10 transformed P-values in human COVID-19 patient lung tissues (SARS-CoV-2) and HCC515 cells with ACE2 inhibitor inhibition (ACE2i), respectively. Size of the bubble shows the average value of -log10 transformed P-value in SARS-CoV-2 and ACE2i.

**Figure 4.**
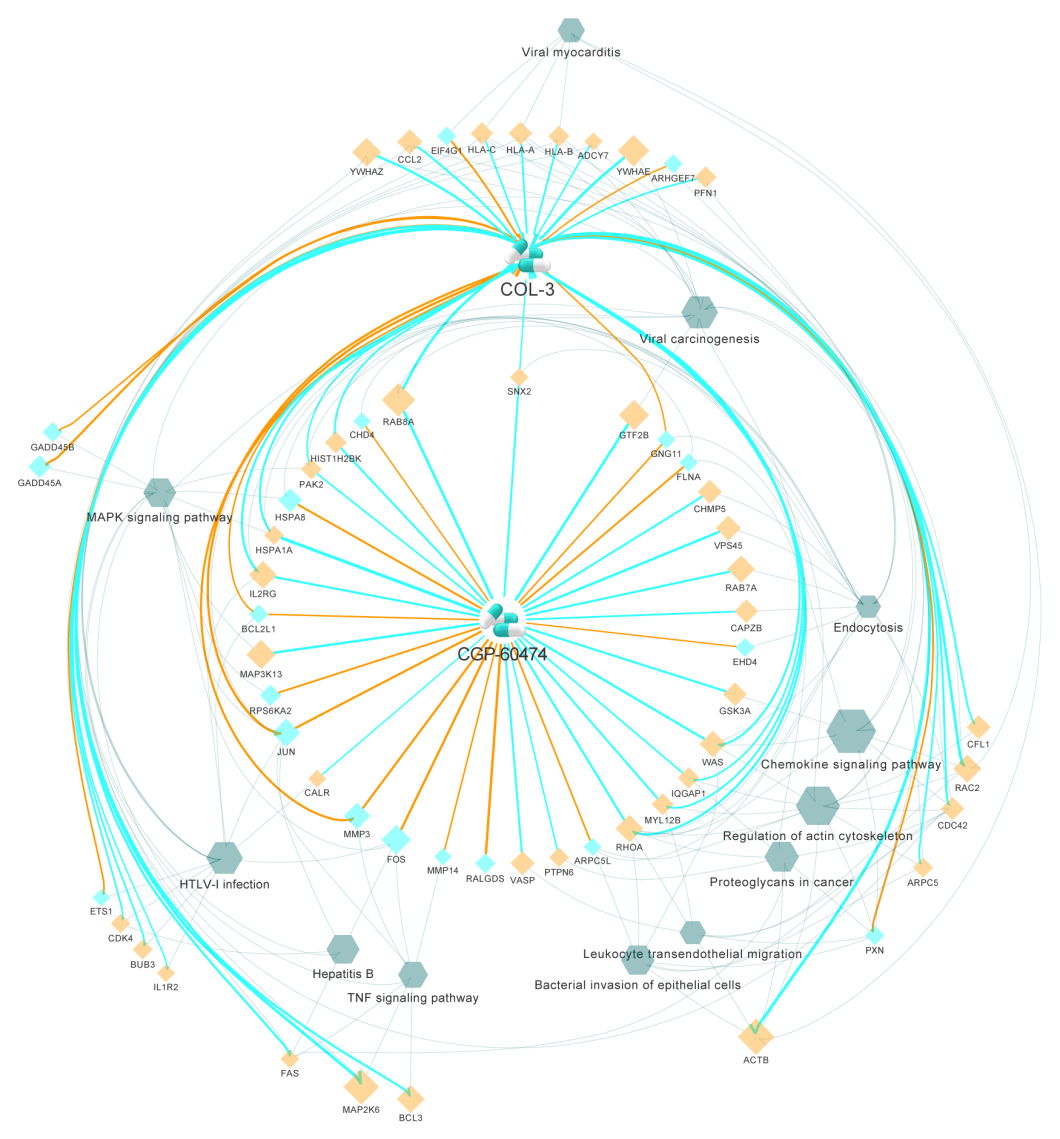
Target genes and pathways of COL-3 and CGP-60474 in treating lung injury in COVID-19. All pathways were significant enriched in both human COVID-19 patient lung tissues and HCC515 cells with ACE2 inhibitor inhibition. The abnormal gene expression patterns in these pathways were reversed by COL-3 and/or CGP-60474. Blue diamond: down-regulated gene in disease; orange diamond: up-regulated gene in disease; hexagon: pathway; blue line: drug decreases gene expression; orange line: drug increases gene expression; blue/orange line width corresponds to the ability to change gene expression; dark green line: interaction between gene and pathway; diamond size: importance of gene in the disease; hexagon size: importance of pathway in the disease.

**Figure 5.**
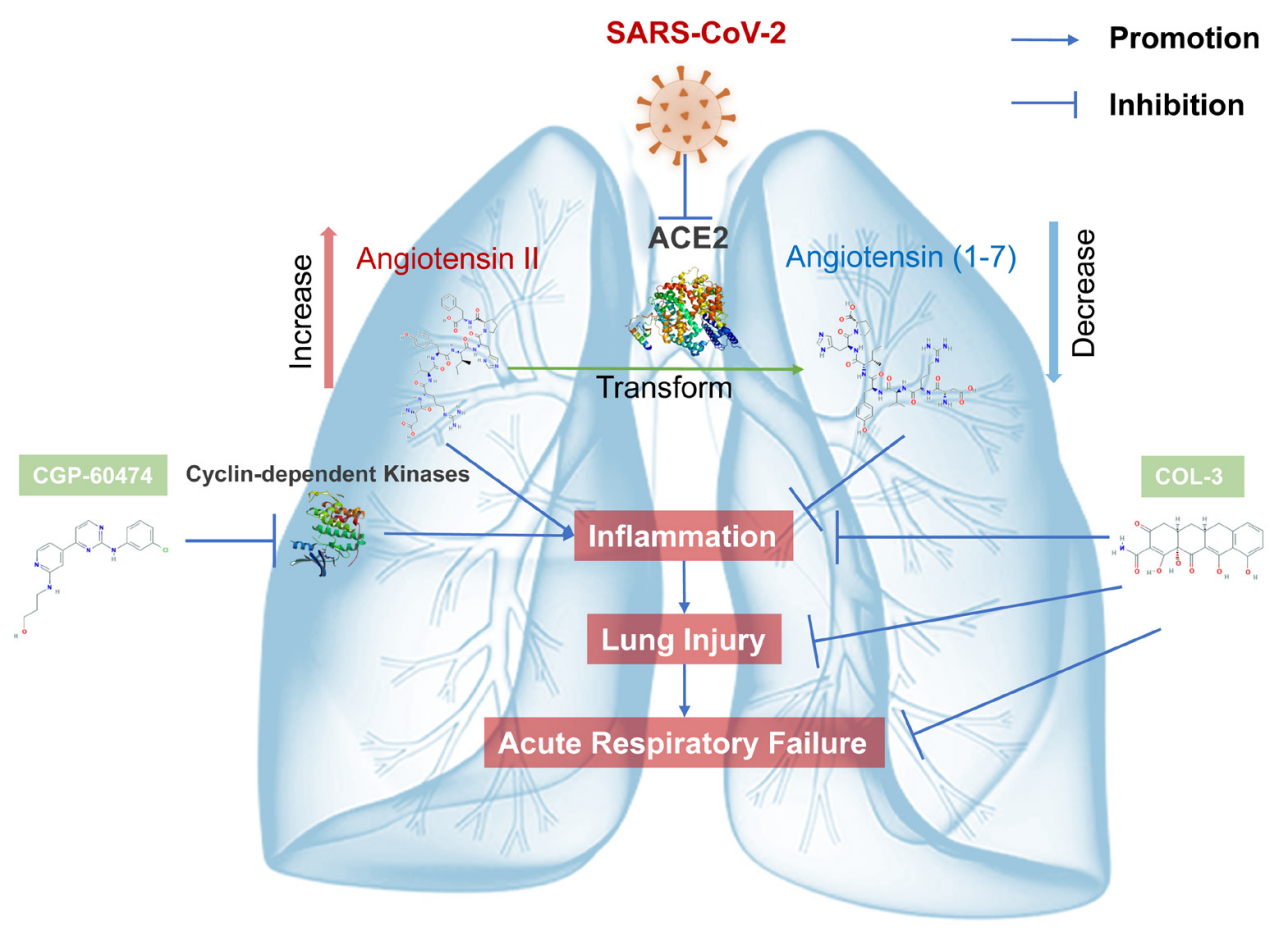
Proposed mechanisms of lung injury in COVID-19 through ACE2 and the therapeutic effects of COL-3 and CGP-60474.

Figure 1 and Figure 5 were generated in Microsoft PowerPoint 2016. Figure 2 and Figure 3 were generated in R (version: 3.6.3) with ggplot2 package (version: 3.3.0) ^18^. Figure 4 was generated in Cystoscope (version: 3.7.2) ^19^.

## Results

### Feasibility test of the drug repositioning pipeline using influenza A (H1N1) infection data

Our drug repositioning is based on the assumption that if a drug can reverse the abnormality of gene expression pattern in the disease, the drug should be able to treat the disease ^20, 21^. Towards this we have implemented the computational framework as shown in Figure 1. We collected differential gene expression patterns in the disease and in cells with drug treatment. Then we searched reversible genes whose expression changes in drug treatment are opposite to those in disease to estimate the effect of a drug for the disease. We further compared effect of every drug to all other candidates to estimate the significance of a drug for treating the disease.

As COVID-19 is an emerging disease with much unknown, we first demonstrate the feasibility of the drug repositioning pipeline using H1N1 virus infection, where much more research has been done and multiple drugs are approved by the United States Food and Drug Administration. We computed the differentially expressed genes from RNA-Seq data of A549 lung cells with or without H1N1 virus infection. We then identified the best candidates that could reverse the expression pattern of these differentially expressed genes, by analyzing 12,706 drugs and compounds from LINCS L1000 pharmacogenomics data ^14^. The results show that CGP-60474 (FDR= 2.514×10 ^-4^), sirolimus (FDR= 3.040×10 ^-4^), COL-3 (FDR= 9.452×10 ^-4^), PIK-75 (FDR= 0.002), geldanamycin (FDR= 0.001), and wortmannin (FDR= 0.046) could significantly (FDR<0.05) reverse the gene expression in H1N1 infection in A549 lung cells ( Table 1). Sirolimus, the second-best candidate by FDR, also known as rapamycin, is a potent immunosuppressant that acts by selectively blocking the transcriptional activation of cytokines, thereby inhibiting cytokine production. It was previously shown clinically effective in H1N1 infected patients with severe pneumonia and acute respiratory failure ^22^ as adjuvant treatment with steroids. PIK-75, a PI3K inhibitor, exhibits potent antiviral activity against H1N1 virus ^23^. Geldanamycin is a Hsp90 inhibitor, while wortmannin is an inhibitor of actinin-4. Both geldanamycin and wortmannin can inhibit H1N1 virus replication ^24, 25^. In summary, our drug repositioning pipeline has shown promise through the example of H1N1 infection.

**Table 1.** Significant candidate drugs for treating infection of H1N1, inhibition of ACE2 and infection of SARS-CoV-2, respectively.

Drug	FDR value			
	H1N1 infection	ACE2i		SARS-CoV-2 infection
	A549 cell	HCC515 cell	HCC515 cell	Human lung tissue
	9h	6h	24h	NA
Sirolimus	3.040×10 ^-4^	NS	NS	0.003
COL-3	9.452×10 ^-4^	NS	0.002	0.003
Geldanamycin	0.001	0.006	NS	NS
CGP-60474	2.514×10 ^-4^	NS	1.337×10 ^-7^	0.003
Staurosporine	NS	NS	NS	0.003
Mitoxantrone	NS	NS	NS	0.003
Trichostatin-a	NS	NS	0.004	NS
Panobinostat	NS	NS	2.443×10 ^-5^	NS
Narciclasine	NS	0.006	NS	NS
PIK-75	0.002	NS	NS	NS
Wortmannin	0.046	NS	NS	NS

NS, not significant; NA, not available; ACE2i, inhibition of ACE2; FDR, false discovery rate.

### Repurposed drugs for treating lung injury in COVID-19

To repurpose drugs for inhibition of ACE2, we conducted differential gene expression analysis in HCC515 and A549 lung cells with the inhibition of ACE2 by moexipril, from the LINCS L1000 project ^14^ using a similar approach as for H1N1 infection described above. Upon examination of ACE2 expression at different time points (six and 24 hours), we opted to focus on HCC515 cells, which have reduced ACE2 expression upon treatment with moexipril, an ACE2 inhibitor. At six hours after treatment with moexipril, narciclasine (FDR=0.006) and geldanamycin (FDR=0.006) could significantly reverse the gene expression changes due to the ACE2 inhibitor ( Table 1). At 24 hours post treatment of moexipril, the effect of CGP-60474 (FDR=1.337×10 ^-7^), panobinostat (FDR=2.443×10 ^-05^), trichostatin-a (FDR=3.546×10 ^-03^) and COL-3 (FDR= 0.002) became significant ( Table 1). Among these predicted drugs, narciclasine and geldanamycin are significant in HCC515 cells at 6h after treatment but no longer significant in cells at 24h after treatment. Both narciclasine and geldanamycin have anti-inflammatory effects and can reduce lung injury caused by other diseases in animal model ^26, 27^. On the other hand, in HCC515 cells treated with moexipril, the ACE2 level at 24h is lower than that at 6h, suggesting that ACE2 inhibition is enhanced over time. Thus drugs such as narciclasine and geldanamycin that are effective in early treatment may not be suitable for sustained administration.

To further confirm if these effects shown in cell lines are physiologically relevant for human lung injury due to COVID-19, we analyzed the RNA-Seq data of human lung tissues from two COVID-19 deceased patients with age-matched normal lung tissues, as reported by Melo *et al.* ^12^ Gene expression of individual markers for lung injury, advanced glycosylation end-product specific receptor (AGER), lipopolysaccharide binding protein (LBP) and secretoglobin family 1A member (SCGB1A1) ^28^ is up-regulated in the HCC515 cell line treated with ACE2 inhibitor and human COVID-19 patient lung tissue ( Figure 2), whereas expression of surfactant protein D (SFTPD), a gene encoding a protein involved in the innate immune response to protect the lungs against inhaled microorganisms and chemicals, is decreased. This indicates the similarity between ACE2 inhibition by moexipril in the cell line and lung injury from COVID-19. Next we extracted the differentially expressed genes in COVID-19 lung tissues vs. normal lungs and used them as target genes to be reversed by the same drugs and compounds in the drug repositioning framework as shown in Figure 1. The results show that sirolimus (FDR=0.003), COL-3 (FDR=0.003), CGP-60474 (FDR=0.003), staurosporine (FDR=0.003) and mitoxantrone (FDR=0.003) are significant in reversing the target genes’ expression in the human lung tissues due to COVID-19 mentioned earlier ( Table 1). Thus, together COL-3 and CGP-60474 show consistent effects for reversing gene expression changes in both the HCC515 cell line treated with ACE2 inhibitor and human COVID-19 patient lung tissue ( Table 1). Moreover, COL-3 and CGP-60474 both can reversely decrease the expression of marker genes for lung injury, AGER, LBP, SCGB1A1, and reversely increase SFTPD expression in HCC515 cell line pre-treated with ACE2 inhibitor moexipril. CGP-60474 (0.12 µM) appears to be more potent than COL-3 (2.5 µM). In conclusion, COL-3 and CGP-60474 show promise as potential purposeful drugs to treat lung injury in COVID-19.

### Pathway comparison between inhibition of ACE2 and infection of SARS-CoV-2

We performed pathway enrichment analysis with the top 1000 deregulated genes in HCC515 cells with ACE2 inhibitor inhibition and human COVID-19 patient lung tissues. It was found that 12 significantly enriched pathways (P-value <0.05) overlap between HCC515 cells with ACE2 inhibitor inhibition and human COVID-19 patient lung tissues ( Figure 3, Table 2). As expected, multiple pathways involved in virus infection are enriched. Various signaling pathways, such as the TNF signaling pathway, MAPK signaling pathway and chemokine signaling pathway, with well-known associations with lung injury, are also enriched ^29– 31^. Moreover, other pathways related to cancers (e.g. ‘viral carcinogenesis’ and ‘proteoglycans in cancer’), or cardiovascular diseases (e.g. ‘viral myocarditis’) also show up significantly enriched in the results ( Figure 3, Table 2). A total of 66 genes in these overlapped pathways show consistent changes between the ACE2 inhibited lung cell line and SARS-CoV-2 lung tissues ( Table 2).

**Table 2.** Pathway comparison between HCC515 cells with ACE2 inhibitor inhibition and human COVID-19 patient lung tissues.

Pathway name	P-value		Consistent genes
	SARS-CoV-2	ACE2i	
	Human lung tissue	HCC515 cell	
Viral carcinogenesis	8.610×10 ^-06^	6.744×10 ^-03^	YWHAZ, PXN, CDC42, HIST1H2BK, RHOA, CHD4, TP53, HLA-A, HLA-C, HLA-B, CDK4, YWHAE, GTF2B, JUN
Endocytosis	4.068×10 ^-05^	3.902×10 ^-02^	RAB7A, CHMP5, SNX2, HSPA1A, ARPC5, CAPZB, CDC42, RHOA, IL2RG, HSPA8, EHD4, RAB8A, VPS45, HLA-A, HLA-C, HLA-B, WAS, ARPC5L, ARF3
Hepatitis B	3.354×10 ^-04^	1.227×10 ^-02^	YWHAZ, TP53, RAF1, CDK4, STAT6, FOS, JUN, FAS
Chemokine signaling pathway	3.797×10 ^-04^	8.760×10 ^-04^	CCL2, ADCY7, GNG11, PXN, CDC42, RAC2, RHOA, RAF1, WAS, GSK3A, GNB1
MAPK signaling pathway	5.283×10 ^-04^	1.257×10 ^-02^	HSPA1A, FOS, CDC42, RAC2, PAK2, FAS, MAP2K6, HSPA8, TP53, NR4A1, RAF1, FLNA, RPS6KA2, JUN, GADD45B, GADD45A, MAP3K13
Regulation of actin cytoskeleton	4.760×10 ^-03^	2.189×10 ^-03^	ARPC5, PXN, IQGAP1, CDC42, PFN1, RAC2, PAK2, RHOA, ACTB, ARHGEF7, RAF1, MYL12B, WAS, ARPC5L, CFL1
Leukocyte transendothelial migration	5.122×10 ^-03^	3.452×10 ^-02^	ACTB, MYL12B, PXN, VASP, CDC42, RAC2, RHOA
Bacterial invasion of epithelial cells	1.697×10 ^-02^	1.130×10 ^-02^	ACTB, CDC42, ARPC5L, RHOA, ARPC5, WAS, PXN
Proteoglycans in cancer	1.870×10 ^-02^	9.963×10 ^-03^	ACTB, PTPN6, TP53, RAF1, IQGAP1, PXN, FLNA, CDC42, RHOA, FAS
TNF signaling pathway	3.117×10 ^-02^	2.039×10 ^-02^	CFLAR, CCL2, MMP14, MMP3, FOS, JUN, BCL3, FAS, MAP2K6
Viral myocarditis	3.976×10 ^-02^	2.943×10 ^-02^	ACTB, EIF4G1, RAC2, HLA-A, HLA-C, HLA-B
HTLV-I infection	4.296×10 ^-02^	7.225×10 ^-03^	IL1R2, ADCY7, BCL2L1, CALR, FOS, IL2RG, BUB3, EGR1, TP53, HLA-A, HLA-C, HLA-B, CDK4, ETS1, JUN

ACE2i, inhibition of ACE2. Consitent genes, whose expression showed same direction (increase or decrease) changes in HCC515 cells with ACE2 inhibitor treatment and lung tissues with SARS-CoV-2 infection.

We further analyzed the genes and pathways associated with the two drugs COL-3 and CGP-60474, which show coherent effects in reversing the gene expression patterns in HCC515 cells with ACE2 inhibitor inhibition and human COVID-19 patient lung tissues ( Figure 4). For COL-3, from the molecular point of view, it leads to decreased expression of many genes including RHOA, RAC2, FAS and CDC42 in lung cells, as part of the mechanisms to protect lung from injury ( Figure 4). These genes are important players in pathways such as the chemokine signaling pathway (for CCL2, ADCY7, GNG11, PXN, CDC42, RAC2, RHOA, WAS), TNF signaling pathway (for CCL2, MMP3, JUN, BCL3, FAS, MAP2K6) and MAPK signaling pathway (for HSPA1A, CDC42, RAC2, PAK2, FAS, MAP2K6, JUN, GADD45B, GADD45A). All 12 significantly enriched pathways in Figure 3 are also observed in COL-3 treatment. CGP-60474 shares 13 gene targets with COL-3, including RHOA, WAS, HSPA1A, SNX2, RAB8A, IL2RG, MMP3, BCL2L1, JUN, HIST1H2BK, GNG11, IQGAP1 and MYL12B. It also has a unique set of target genes related to lung injury, such as CALR and MMP14 ( Figure 4). It decreases the expression of CALR, a multifunctional protein that acts as a major Ca(2+)-binding (storage) protein in the lumen of the endoplasmic reticulum ^32^. It also increases the expression of MMP14, a member of the matrix metalloproteinase (MMP) family with anti-inflammatory properties. CGP-60474 treatment affects 11 out of 12 significantly enriched pathways in COL-3, but not the viral myocarditis pathway. More details on the molecular mechanisms of the target genes and pathways of these two drug candidates are discussed below.

## Discussion

The inhibition of ACE2 promotes lung injury via the renin–angiotensin system (RAS) ^33^. In pulmonary RAS, ACE2 converts angiotensin II (Ang II), an octapeptide hormone, to Ang-(1-7), an heptapeptide hormone ( Figure 5). Ang II triggers pulmonary inflammation and activates the TNF signaling pathway and MAPK signaling pathway to promote lung injury ^34, 35^. On the other hand, Ang-(1–7) inhibits inflammation and protects lungs from injury ^36^ by inhibiting the MAPK signaling pathway ^37^, lowering cytokine release ^38^ and downregulating the RHOA/ROCK pathway ^39^. Thus, inhibition of ACE2 will increase Ang II levels, decrease Ang-(1–7), and deregulate various downstream pathways, such as TNF and MAPK signaling pathways to promote lung injury ( Figure 5). Our pathway analysis on the HCC515 lung cell line confirmed that inhibition of ACE2 by moexipril can deregulate TNF signaling, MAPK signaling and cytokine signaling pathways. We further showed that these pathways are also deregulated in human lung tissues of deceased COVID-19 patients ( Table 2). Moreover, inhibition of ACE2 induced similar expression patterns of lung injury markers to that in human lung tissues of deceased COVID-19 patients ( Figure 2). This evidence suggests that inhibition of ACE2 may indeed be part of the molecular mechanisms of lung injury in COVID-19. Moreover, other pathways related to cancers (e.g. ‘viral carcinogenesis’ and ‘proteoglycans in cancer’), or cardiovascular diseases (e.g. viral myocarditis) also show up significantly enriched in the results ( Table 2). These results may help to explain the increased risks of fatality among COVID-19 patients with underlying conditions (cancers, heart diseases) ^40, 41^. Additionally, myocarditis has been clinically observed in a patient with COVID-19 ^42^, showing a direct link between the two conditions.

Our drug repositioning analysis suggested five possible drugs based on RNA-Seq data from patients deceased from COVID-19. Among them, clinical trial has started for treating patients with COVID-19 pneumonia with sirolimus ( NCT04341675). Two other drugs (or compounds), COL-3 and CGP-60474, also have additional evidence of effectiveness from the L1000 data of the lung HCC515 cell line treated with ACE2 inhibitor moexipril. Moreover, both COL-3 and CGP-60474 could reverse the expression patterns of lung injury markers in HCC515 cells with ACE2 inhibitor inhibition and human COVID-19 patient lung tissues ( Figure 2). This phenotypic evidence suggests that COL-3 and CGP-60474 may be effective in treating lung injury in COVID-19 ( Figure 5). Therefore, we further analyzed the target genes and pathways of these two drugs in treating lung injury in COVID-19. 

COL-3, also known as incyclinide or CMT-3, is a chemically modified tetracycline. It reversed the expression patterns of many lung injury related genes and pathways, such as RHOA, RAC2 and FAS in the chemokine signaling pathway, TNF signaling pathway and MAPK signaling pathway ( Figure 4). RHOA, also known as ras homolog family member A, is a member of the Rho family of small GTPases. The activation of RHOA is crucial for lung injury ^43^. Inhibition of RHOA is a promising approach to acute lung injury treatment ^44^. RAC2, also known as Ras-related C3 botulinum toxin substrate 2, is a member of the Ras superfamily of small guanosine triphosphate (GTP)-metabolizing proteins. Rac2 plays an important role in inflammation-mediated lung injury ^45, 46^. FAS, also known as Fas cell surface death receptor, is a member of the TNF-receptor superfamily. FAS activation is essential in inducing acute lung injury ^47^. Small interfering RNA targeting Fas reduced lung injury in mice ^48^. Previous results from many pre-clinical animal models have supported the role of COL-3 in reducing lung injury and improves survival of experimented animals. For example, COL-3 prevented lung injury and acute respiratory distress syndrome (ARDS) in a clinically applicable porcine model ^49– 55^. It also improved acute respiratory distress syndrome (ARDS) survival in an ovine model ^56^. Given all the evidence, COL-3 may be an attractive candidate for a clinical trial treating severe viral pneumonia related lung injury with respiratory failure in COVID-19 ( Figure 5).

CGP-60474, on the other hand, is an inhibitor of cyclin-dependent kinase ( Figure 5). CGP-60474 not only shared target genes with COL-3, such as RHOA, WAS, HSPA1A, SNX2, RAB8A, IL2RG, MMP3, BCL2L1, JUN, HIST1H2BK, GNG11, IQGAP1 and MYL12B, but also has unique target genes that related to lung injury, like CALR and MMP14 ( Figure 4). Blocking CALR activity attenuated murine acute lung injury by inducing polarization of M2 subtype macrophages, which are anti-inflammatory ^57^. MMP14 was shown to trigger the anti-inflammatory proteolytic cascade to prevent lung injury in mice ^58^. Interestingly, so far only a few studies have reported some biological functions of CGP-60474 ^59– 61^. One drug reposition study using L1000 data also pointed to CGP-60474 as the most potent drug based on anti-inflammatory effects ^61^. The authors then experimentally showed that CGP-60474 alleviated tumor necrosis factor-α (TNF-α) and interleukin-6 (IL-6) levels in activated macrophages, downregulated the NF-κB activity, and reduced the mortality rate in lipopolysaccharide induced endotoxemia mice. Another *in silico* drug prediction study suggested that CGP-60474 could target multiple cancers, though no experiments were conducted ^59^. Although cyclin-dependent kinase inhibition by another drug, seliciclib, reduced lung damage in a mouse model of ventilator-induced lung injury ^60^, further *in vivo* investigation of CGP-60474 is needed to test its role in treating lung injury.

In summary, we propose two candidate drugs, COL-3 and CGP-60474, which can reverse the gene expression patterns in COVID-19 lung injury and a lung cell line with ACE2 being inhibited. We further analyzed potential molecular and biological mechanisms of lung injury in COVID-19. The work will hopefully gain the interest of the biomedical and clinical community for further validations *in vivo* for both candidate drugs, and even possibly clinical trials on COL-3 to save lives from severe respiratory failure in COVID-19.

## Data availability

### Source data

RNA-Seq data from Gene Expression Omnibus, Accession number GSE147507: https://identifiers.org/geo:GSE147507

Phase I LINCS L1000 data from Gene Expression Omnibus, Accession number GSE92742: https://identifiers.org/geo:GSE92742

Phase II LINCS L1000 data from Gene Expression Omnibus, Accession number GSE70138: https://identifiers.org/geo:GSE70138

### Underlying data

Zenodo: lanagarmire/COVID19-Drugs-LungInjury: Prediction of repurposed drugs for treating lung injury in COVID-19. https://doi.org/10.5281/zenodo.3823277 ^62^

This project contains the following underlying data:

- HCC515_6_data_for_drug.csv (Differential expression of genes in HCC515 cell at 6 h after treatment of ACE2 inhibitor)
- HCC515_24_data_for_drug.csv (Differential expression of genes in HCC515 cell at 24 h after treatment of ACE2 inhibitor)
- COVID19-Lung_data_for_drug.csv (Differential expression of genes in lung tissues with COVID-19)
- HCC515_6_drug.csv (Drugs for HCC515 cell at 6 h after transfection of ACE2 inhibitor)
- HCC515_24_drug.csv (Drugs for HCC515 cell at 24 h after transfection of ACE2 inhibitor)
- COVID19-Lung_drug.csv (Drugs for lung tissuse from COVID-19 patients)
- COL-3_single_treatment_response_data.csv (Differential expression of genes in HCC515 cell at 24h after treatment of COL-3)
- CGP-60474_single_treatment_response_data.csv (Differential expression of genes in HCC515 cell at 24h after treatment of CGP-60474)

Data are available under the terms of the Creative Commons Attribution 4.0 International license (CC-BY 4.0).

### Code availability

Source code available from: https://github.com/lanagarmire/COVID19-Drugs-LungInjury

Archived source code at time of publication: https://doi.org/10.5281/zenodo.3822923 ^13^

License: GNU General Public License v3.0
