# Supplementary material for: Prediction of repurposed drugs for treating lung injury in COVID-19
Source: F1000Res. 2020 Aug 26;9:609. Originally published 2020 Jun 15. [Version 2] doi: 10.12688/f1000research.23996.2 (PMC7468567; doi:10.12688/f1000research.23996.2)
Supplement: Peer review history

# Peer review: Reviewer response for version 2

**DOI:** 10.5256/f1000research.28930.r70268

**Published:** 2020-09-02

**Authors:** Xia Yang

**Affiliations:** 1 Department of Integrative Biology and Physiology, University of California, Los Angeles, Los Angeles, CA, USA

The authors have sufficiently addressed my comments.

Is the work clearly and accurately presented and does it cite the current literature?

Yes

If applicable, is the statistical analysis and its interpretation appropriate?

Partly

Are all the source data underlying the results available to ensure full reproducibility?

Yes

Is the study design appropriate and is the work technically sound?

Partly

Are the conclusions drawn adequately supported by the results?

Yes

Are sufficient details of methods and analysis provided to allow replication by others?

Yes

Reviewer Expertise:

Bioinformatics, systems biology, complex diseases

I confirm that I have read this submission and believe that I have an appropriate level of expertise to confirm that it is of an acceptable scientific standard.

# Peer review: Reviewer response for version 2

**DOI:** 10.5256/f1000research.28930.r70269

**Published:** 2020-08-26

**Authors:** Leng Han, Shengli Li

**Affiliations:** 1 Department of Biochemistry and Molecular Biology, The University of Texas Health Science Center at Houston McGovern Medical School, Houston, TX, USA

I have no further comments to make.

Is the work clearly and accurately presented and does it cite the current literature?

Yes

If applicable, is the statistical analysis and its interpretation appropriate?

Yes

Are all the source data underlying the results available to ensure full reproducibility?

Yes

Is the study design appropriate and is the work technically sound?

Yes

Are the conclusions drawn adequately supported by the results?

Yes

Are sufficient details of methods and analysis provided to allow replication by others?

Yes

Reviewer Expertise:

Computational biology

We confirm that we have read this submission and believe that we have an appropriate level of expertise to confirm that it is of an acceptable scientific standard.

# Peer review: Reviewer response for version 1

**DOI:** 10.5256/f1000research.26472.r65750

**Published:** 2020-07-10

**Authors:** Leng Han, Shengli Li

**Affiliations:** 1 Department of Biochemistry and Molecular Biology, The University of Texas Health Science Center at Houston McGovern Medical School, Houston, TX, USA

He *et al.* implemented a computational framework of drug repositioning to identify drug candidates for COVID-19 lung injury treatment. The authors first proved feasibility of drug repositioning pipeline in H1N1 infection data. They extracted genes that showed differential expression in ACE2 inhibitor-treated cell line and COVID-19 patients. The authors next screened drugs that could reverse expression of these differential genes, wherein COL-3 and CGP-60474 showed the most potent to treat lung injury in COVID-19.

1. In the moexipril-treated HCC515 cell line, the effects of narciclasine and geldanamycin disappeared at 24 hours post treatment, which could significantly reverse the gene expression changes at six hours after treatment. Please provide more discussion on this.
2. How many genes (key genes) were deregulated in the same direction in both ACE2 inhibition-treated HCC515 cell line and COVID-19 patient lung tissue?
3. For referring drug candidates, did you use all the differentially expressed genes in COVID-19 lung tissues vs. normal lungs, or only part of differential genes?
4. In Table 1, column "SARS-CoV-2 infection Human lung tissue", the P values of effective drugs are the same, please provide more details to explain.

Is the work clearly and accurately presented and does it cite the current literature?

Yes

If applicable, is the statistical analysis and its interpretation appropriate?

Yes

Are all the source data underlying the results available to ensure full reproducibility?

Yes

Is the study design appropriate and is the work technically sound?

Yes

Are the conclusions drawn adequately supported by the results?

Yes

Are sufficient details of methods and analysis provided to allow replication by others?

Yes

Reviewer Expertise:

Computational biology

We confirm that we have read this submission and believe that we have an appropriate level of expertise to confirm that it is of an acceptable scientific standard.

# Response

**Published:** 2020-08-17

**Authors:** Bing He

**Affiliations:** University of Michigan, USA

He *et al.* implemented a computational framework of drug repositioning to identify drug candidates for COVID-19 lung injury treatment. The authors first proved feasibility of drug repositioning pipeline in H1N1 infection data. They extracted genes that showed differential expression in ACE2 inhibitor-treated cell line and COVID-19 patients. The authors next screened drugs that could reverse expression of these differential genes, wherein COL-3 and CGP-60474 showed the most potent to treat lung injury in COVID-19.

- In the moexipril-treated HCC515 cell line, the effects of narciclasine and geldanamycin disappeared at 24 hours post treatment, which could significantly reverse the gene expression changes at six hours after treatment. Please provide more discussion on this.

**Response: **Thank you for your comment. We have added more discussion on this according to your suggestion.

"Among these predicted drugs, narciclasine and geldanamycin are significant in HCC515 cells at 6h after treatment but no longer significant in cells at 24h after treatment. Both narciclasine and geldanamycin have anti-inflammatory effects and can reduce lung injury caused by other diseases in animal model. On the other hand, in HCC515 cells treated with moexipril, the ACE2 level at 24h is lower than that at 6h, suggesting that ACE2 inhibition is enhanced over time. Thus drugs such as narciclasine and geldanamycin that are effective in early treatment may not be suitable for sustained administration.”

 

- How many genes (key genes) were deregulated in the same direction in both ACE2 inhibition-treated HCC515 cell line and COVID-19 patient lung tissue?

 

**Response: **There are 5390 genes deregulated in the same direction in both ACE2 inhibition-treated HCC515 cell line and COVID-19 patient lung tissue. Among them, 797 genes are in top 1000 differentially expressed genes in either ACE2 inhibition-treated HCC515 cell line or COVID-19 patient lung tissue, and 119 genes are in significantly enriched pathways. We have added these statistics into the manuscript according to your comment.

 

- For referring drug candidates, did you use all the differentially expressed genes in COVID-19 lung tissues vs. normal lungs, or only part of differential genes?

**Response: **We used all differentially expressed genes in drug repurposing analysis.

 

- In Table 1, column "SARS-CoV-2 infection Human lung tissue", the P values of effective drugs are the same, please provide more details to explain.

**Response:** Good question. Table 1 shows BH FDR adjusted P value for every drug. The raw p value for the top 5 drugs are 5.11e-7,6.38e-7,9.02e-7,1.21e-6 and 1.34e-6. There are 12706 drugs. Since FDR=(Number of drugs x P.value)/Drug rank , FDR for the top 5 drugs are 0.00649, 0.00405,0.00382,0.00385 and 0.00340. According to BH procedure, if FDR of a drug is bigger than the FDR of the next lower ranked drug, the FDR of that drug is reset to the FDR of next lower ranked drug. So the FDR for the top 4 drugs are set to the FDR of the fifth drug, which is 0.00340. This is a conservative measure. That’s why FDR of all top 5 drugs are 0.00340, which is rounded to 0.003 in table 1.

# Peer review: Reviewer response for version 1

**DOI:** 10.5256/f1000research.26472.r64847

**Published:** 2020-06-30

**Authors:** Xia Yang

**Affiliations:** 1 Department of Integrative Biology and Physiology, University of California, Los Angeles, Los Angeles, CA, USA

In this study, He and Garmire utilized publicly available gene expression datasets on COVID-19 lungs and drug treatment studies in lung cell lines to conduct drug repositioning analysis. They found that the genes and pathways altered by an AEC2 inhibitor in the HCC515 lung cell line mimic those of COVID-19 lungs, supporting that ACE2 inhibition likely underlines the lung injury induced by the SARS-CoV2 virus. They used the gene profiles from these datasets to identify drugs in the L1000 database that can reverse the genes and pathways affected by ACE2 inhibition and in COVID-19 lungs and prioritized two drugs, COL-3 and CGP-60474, that have such properties and may have the potential to treat COVID-19. The study was well-designed and executed, the paper was well-written and easy to follow, and the findings are interesting and promising. The genes, pathways, and drugs uncovered offer insights into COVID-19. Overall a very timely study to address a critical health issue. There are some technical aspects to be addressed, as detailed below.

 Major:

1. Some of the statistical analyses need justification. The arbitrary use of top 1000 genes instead of any statistical cutoff is unconventional. Pathway analysis used raw p values instead of FDRs, which is also unconventional. Please justify.
2. Unclear what type of genes are considered in n_consistent in the pathway score calculation. Among top 1000 genes in both ACE2i and COVID19? If so, state it clearly.
3. As many curated pathways that are closely related (e.g., inflammation pathways) share overlapping genes, they tend to show coordinated over- or under-enrichment in pathway analysis. The use of n_pathway in the gene score calculation can be misleading due to such overlaps in non-independent pathways.
4. Which method was used in the FDR calculation for drug repositioning analysis? Please clarify.
5. Datasets related to H1N1 was used as a positive control to test the drug repositioning pipeline since numerous FDA-approved drugs are available. This is a good design but only one of the drugs predicted was discussed. What about the other top ranked drugs? Any evidence for H1N1 treatment effect for the other top ranked drugs? What about the FDR approved drugs for H1N1? Were they retrieved by the analysis? These are unclear based on the results descriptions. If not, the conclusion "our drug repositioning pipeline has shown merit in discovering effective drugs through the example of H1N1 infection" is too strong.
6. For genes in Table 2, not sure if the direction of changes is consistent. Please clarify.

Minor:

1. Add year to the date "12th May".
2. It is interesting that the ACE2 inhibitor only inhibited ACE2 in HCC515 cell line but not the A549 cell line. Any explanation on why the drug did not inhibit ACE2 in A549?

Is the work clearly and accurately presented and does it cite the current literature?

Yes

If applicable, is the statistical analysis and its interpretation appropriate?

Partly

Are all the source data underlying the results available to ensure full reproducibility?

Yes

Is the study design appropriate and is the work technically sound?

Partly

Are the conclusions drawn adequately supported by the results?

Yes

Are sufficient details of methods and analysis provided to allow replication by others?

Yes

Reviewer Expertise:

Bioinformatics, systems biology, complex diseases

I confirm that I have read this submission and believe that I have an appropriate level of expertise to confirm that it is of an acceptable scientific standard, however I have significant reservations, as outlined above.

# Response

**Published:** 2020-08-17

**Authors:** Bing He

**Affiliations:** University of Michigan, USA

In this study, He and Garmire utilized publicly available gene expression datasets on COVID-19 lungs and drug treatment studies in lung cell lines to conduct drug repositioning analysis. They found that the genes and pathways altered by an AEC2 inhibitor in the HCC515 lung cell line mimic those of COVID-19 lungs, supporting that ACE2 inhibition likely underlines the lung injury induced by the SARS-CoV2 virus. They used the gene profiles from these datasets to identify drugs in the L1000 database that can reverse the genes and pathways affected by ACE2 inhibition and in COVID-19 lungs and prioritized two drugs, COL-3 and CGP-60474, that have such properties and may have the potential to treat COVID-19. The study was well-designed and executed, the paper was well-written and easy to follow, and the findings are interesting and promising. The genes, pathways, and drugs uncovered offer insights into COVID-19. Overall a very timely study to address a critical health issue. There are some technical aspects to be addressed, as detailed below.

Major:

1. Some of the statistical analyses need justification. The arbitrary use of top 1000 genes instead of any statistical cutoff is unconventional. Pathway analysis used raw p values instead of FDRs, which is also unconventional. Please justify.

**Response:** Thank you for your comments. We got gene expression data in response to AEC2 inhibitor in the HCC515 lung cell line from LINC1000 project. It only provides differential gene expression levels measured in Z-score. So we can only select differential genes by using top n genes. To be consistent, in this study, we also transformed all differential expressions in Z-score and used top n to select differentially expressed genes. Top 1000 differentially expressed genes in HCC515 cell with AEC2 inhibitor treatment and COVID-19 lungs mapped to 43 and 54 kegg pathways, respectively. The FDR for the most significantly pathway in HCC515 cell with AEC2 inhibitor treatment is 0.21. If we use FDR instead of p value in pathway analysis, then no further analysis could be performed in this study.

 

- Unclear what type of genes are considered in n_consistent in the pathway score calculation. Among top 1000 genes in both ACE2i and COVID19? If so, state it clearly.

**Response:** Thank you for the comment. We have revised the manuscript accordingly. “ *n_ _consistent_*is the number of consistent genes in that pathway among top 1000 differential expressed genes, for both HCC515 cells with ACE2 inhibitor treatment and lung tissues of deceased COVID-19 patients”.

 

- As many curated pathways that are closely related (e.g., inflammation pathways) share overlapping genes, they tend to show coordinated over- or under-enrichment in pathway analysis. The use of n_pathway in the gene score calculation can be misleading due to such overlaps in non-independent pathways.

**Response:** Thank you for your comment. We removed n_pathway from gene score calculation according to your suggestion, and updated figure 4 according to new gene score.

 

- Which method was used in the FDR calculation for drug repositioning analysis? Please clarify.

**Response: **The FDR calculation used Benjamini-Hochberg (BH) procedure. We have clarified this in the manuscript according to your comment.

 

- Datasets related to H1N1 was used as a positive control to test the drug repositioning pipeline since numerous FDA-approved drugs are available. This is a good design but only one of the drugs predicted was discussed. What about the other top ranked drugs? Any evidence for H1N1 treatment effect for the other top ranked drugs? What about the FDR approved drugs for H1N1? Were they retrieved by the analysis? These are unclear based on the results descriptions. If not, the conclusion "our drug repositioning pipeline has shown merit in discovering effective drugs through the example of H1N1 infection" is too strong.

**Response:** Thank you for your comments. Our drug response data in lung cells are collected from LINC1000 project, which unfortunately do not include treatment using current four FDA approved drugs (peramivir, zanamivir, oseltamivir phosphate, and baloxavir marboxil) for H1N1. We have toned down the statement to “our drug repositioning pipeline has shown promise through the example of H1N1 infection". We also added discussion of other top ranked drugs and revised the conclusion according to your suggestions. 

 

- For genes in Table 2, not sure if the direction of changes is consistent. Please clarify.

**Response:** Yes, all genes listed in table 2 have same direction in both HCC515 cell with AEC2 inhibitor treatment and COVID-19 lungs. We have added the annotation to the table 2.

Minor:

- Add year to the date "12th May".

**Response:** We added the year according to your suggestion.

- It is interesting that the ACE2 inhibitor only inhibited ACE2 in HCC515 cell line but not the A549 cell line. Any explanation on why the drug did not inhibit ACE2 in A549?

**Response:** The ACE2 inhibitor inhibited ACE2 in both HCC515 cell and A549 cell. But in A549 cell, Z-score of ACE2 at 24h after treatment is higher than that at 6h, rather than being lower as expected (if ACE2 inhibitor is effective). This implies the inhibitor is not effective in A549 cell for some reason. Due to the concern of data quality, we didn’t use data from A549 cell.
